# Effect of illness perception on self-regulatory fatigue in older adult patients with hypertension: chain-mediating role of self-efficacy and coping style

**DOI:** 10.3389/fpubh.2025.1728453

**Published:** 2025-12-17

**Authors:** Tingting Lu, Lihua Shi, Huijun Zhang, Jianfang Zhang, Yiqing Shen

**Affiliations:** 1 Department of Cardiothoracic Surgery, The Affiliated Suzhou Hospital of Nanjing Medical University, Suzhou Municipal Hospital, Suzhou, China; 2 Department of Nursing, The Affiliated Suzhou Hospital of Nanjing Medical University, Suzhou Municipal Hospital, Suzhou, China; 3 Department of Nursing, Jinzhou Medical University, Jinzhou, China; 4 Department of Emergency and Critical Care Medicine, The Affiliated Suzhou Hospital of Nanjing Medical University, Suzhou Municipal Hospital, Suzhou, China

**Keywords:** aged, hypertension, self efficacy, adaptation, self-regulatory fatigue

## Abstract

**Purpose:**

Long-term self-control becomes necessary for older adult patients with hypertension to sustain their blood pressure levels and postpone disease progression. Over extended periods, such self-control capacity among patients experiences gradual depletion, which leads to self-regulatory fatigue. Nevertheless, the connection linking disease perception, self-efficacy, coping style, and self-regulatory fatigue remains unexplored in existing studies. The present study sought to examine how disease perception, self-efficacy, and coping style relate to self-regulatory fatigue among older adult patients with hypertension.

**Methods:**

Convenience sampling method enabled the recruitment of 416 older adult patients with hypertension from the First Affiliated Hospital of Jinzhou Medical University, Liaoning Province, for this cross-sectional survey. Multiple instruments served as measurement tools, including the general demographic questionnaire, brief illness perception questionnaire, general self-efficacy scale, medical coping modes questionnaire, and self-regulatory fatigue scale. Amos23.0 software facilitated the analysis of the mediation effect.

**Results:**

Self-regulatory fatigue receives direct positive influence from disease perception, avoidance, and acceptance-resignation, whereas self-efficacy and confrontation exert direct negative influence upon it. The impact of illness perception on self-regulatory fatigue operates via chain mediating pathways involving self-efficacy, confrontation, avoidance, and acceptance-resignation.

**Conclusion:**

The older adult patients with hypertension had higher self-regulatory fatigue level. Positive correlations emerged between self-regulatory fatigue and disease perception, avoidance, as well as acceptance-resignation; diminishing patients’ negative emotions toward disease alongside enhancing their treatment confidence contributes to lowering patients’ self-regulatory fatigue.

## 1 Introduction

The main risk factor for cerebrovascular diseases is hypertension. As age increases, the incidence of hypertension continues to rise. Research showed that among Chinese older adults over 65 years old, the prevalence of hypertension is as high as 66.9%, seriously threatening the health and life of the older adult patients (1, 2). Studies have shown that aging causes several physiological changes, such as increased pulse stiffness and expanded pulse pressure, which impact blood pressure and lead to related complications (3). Even though self-management helps control blood pressure and prevent complications, older Chinese adults with hypertension show poor self-management skills, particularly when it comes to managing their exercise (4). This also showed that the ability to exercise self-control was relatively reduced in older adults with hypertension. Baumeister et al. (5) developed the idea of ego-depletion, also known as self-regulatory fatigue, based on the self-control resource theory. According to his theory, ego-depletion was the exhaustion of limited mental resources brought on by personal self-control behaviors, which could result in a range of behavioral, emotional, and cognitive issues (5). Some researchers highlighted that self-regulatory fatigue was a chronic fatigue state that developed after excessive self-control resource use, a type of psychological resource drain that occurs when patients deal with illness-related issues for extended periods of time and from which patients struggle to recover (6). Previous research has demonstrated that the majority of patients with chronic illnesses experience self-regulatory fatigue as a common psychological phenomenon. The patients’ self-control resources were quickly depleted as they endured significant physical and psychological strain while managing their emotions and enduring the discomfort of physical illness (7). Therefore, it becomes crucial to recognize and treat self-regulatory fatigue in older adult hypertension patients in order to reduce psychological burden and improve treatment results.

A person’s perspective on a disease, including patient experiences, ideas, and beliefs about it, is referred to as their illness perception. Patients who describe their current state of illness more negatively score higher (8). Related research has shown that disease cognition is a key factor in behavior guidance in disease management, and that patients may experience health behavior disturbances as a result of incorrect disease cognition (9). According to some researchers, patients easily experienced negative disease experiences while undergoing treatment, which led to erroneous disease perceptions, which in turn caused negative emotional states and impaired behavioral control (10). Therefore, while developing strategies to improve patients’ self-regulatory capacity and reduce their self-regulatory fatigue, nursing staff should expeditiously facilitate accurate disease knowledge dissemination and alleviate negative disease-related emotions.

Self-efficacy is a person’s belief in their own ability to complete a task (11). Yu et al. (12) found that self-efficacy increases one’s capacity for self-control, which in turn promotes physical activity. Therefore, patients with higher levels of self-efficacy also have a comparatively stronger capacity for self-control, and their self-regulatory fatigue intensity decreases as they use comparatively fewer psychological resources to deal with disease-related challenges. The most noticeable symptom among cancer patients, according to Kurt and Altan (13), is fatigue, which gets less severe as patient self-efficacy increases. This implies that increasing self-efficacy may help patients feel less fatigued from adaptation.

One essential psychological tool that helps people deal with stress is their coping style. It plays an important role between the stressor and the stress response in stressful situations (14). According to earlier research, patients can attain a perception of disease control by using healthy coping strategies that help them manage their emotions as well as difficulties and dangers (15). Patients who use constructive coping strategies experience less psychological strain, use fewer psychological resources, and experience less fatigue (16). Coping strategies influence self-control and health outcomes, according to researchers; people who use avoidant coping tend to have poor self-control (17). Therefore, self-regulatory fatigue intensity often increases when patients use negative coping strategies like avoidance and acceptance-resignation. This proved that self-regulatory fatigue was significantly influenced by coping style.

The self-regulatory model was put forth by scholar Leventhal et al. (18). When faced with disease threats, this model accurately depicts the ongoing self-regulatory process patients engage in to preserve their health and reduce the negative effects of their illness. According to the model, people’s coping mechanisms when confronted with health risks depend on how they perceive the threat and their personal beliefs. They also assess the strategies and behaviors they have adopted, and based on the results of their evaluations, they adjust their disease cognition and coping mechanisms (19). In this study, having hypertension was considered a health threat faced by individuals. The illness perception, coping strategies, and self-evaluations experienced by patients constitute their self-regulation processes in response to the threat posed by hypertension. Specifically, illness perception were defined as patients’ perceptions of hypertension, beliefs about the disease treatment process, and beliefs about managing the disease themselves; coping is defined as the ways in which patients respond to the disease; and self-evaluation was defined as the emotional outcomes (self-regulatory fatigue) produced by patients during the self-regulatory process.

Within the present study, the self-regulatory model served as the theoretical foundation for variable hypotheses. Disease perception, self-efficacy, and coping style were regarded as potential factors influencing self-regulatory fatigue for constructing a structural equation model, with the assumption that illness perception could influence self-regulatory fatigue via chain mediation involving self-efficacy and coping style. By verifying the above hypotheses, further analyze the pathways of various influencing factors to provide a theoretical basis for formulating corresponding intervention measures. This study combined the self-regulatory model, focusing on self-regulatory fatigue in older adults patients with hypertension and exploring its related mechanisms, addressing the shortcomings of previous research in terms of theoretical mechanisms.

## 2 Materials and methods

### 2.1 Design and participants

The Institutional Review Board of the Jinzhou Medical University granted approval for the present study (number: JZMULL2023047). Data collection spanned November 2023 through March 2024, with ethical committee approval secured in September 2023. The present study adopted a cross-sectional design, and written strictly in accordance with the STROBE guidelines for cross-sectional studies, ensuring a certain level of comprehensiveness and accuracy. This study used a convenience sampling method to select research subjects. The researchers personally contacted the heads of the Cardiology and Geriatrics departments at the First Affiliated Hospital of Jinzhou Medical University, and after obtaining their consent, formed a research team to collect the data. All participants had a hypertension diagnosis issued by a clinical physician, and the data collection was conducted after the patients had completed treatment and their condition was stable. Subject enrollment requirements comprised: (1) According to the hypertension diagnostic criteria proposed in the 2024 Chinese guidelines for the management of hypertension (20): individuals with a systolic blood pressure ≥140 mmHg or a diastolic blood pressure ≥90 mmHg who are not using antihypertensive drugs. (2) Participants whose age reached 60 years or beyond. (3) Participants possessing consciousness and normal communication capability. (4) Participants capable of providing informed consent and willing to engage in the study voluntarily. Exclusion parameters encompassed: (1) Participants experiencing hypertension accompanied by severe cardiovascular, brain, liver, or kidney dysfunction. (2) Patients carrying diagnoses of mental illness and cognitive impairment.

Following Kendall multivariate analysis sample size specifications, influencing factor studies necessitate sample sizes representing minimally 5–10 times variable quantity (21). The present study incorporated 16 variables total and accounted for 20% attrition, yielding required sample sizes spanning 96–192 cases. Furthermore, structural equation model construction demands sample sizes reaching minimally 200 cases (22). To strengthen research subject representativeness and guarantee result reliability, 416 cases ultimately comprised the sample size for the present study.

### 2.2 General demographic questionnaire

Through group discussion and Literature review, researchers established that general demographic data content primarily encompassed nine elements: age, gender, education level, occupation, marital status, per capita monthly income of the family, number of years of hypertension, number of comorbidities, and chronic pain.

### 2.3 Self-regulatory fatigue scale (SRF-S)

Nes et al. (6) originally developed this scale, with Wang et al. (23) completing its Chinese translation. The Chinese version encompassed 16 items across 3 dimensions. Specifically, cognitive control comprised 6 items, emotional control contained 5 items, and behavior control incorporated 5 items. Employing Likert 5-level scoring methodology, scores ranged from 1 to 5 spanning strongly disagree to strongly agree, wherein items 1, 2, 5, 9, and 14 required reverse scoring, yielding total scores between 16 and 80. Elevated scores signified intensified self-regulated fatigue levels among patients. The present study obtained a Cronbach’s *α* coefficient of 0.96 for the total scale.

### 2.4 Brief illness perception questionnaire (BIPQ)

Broadbent et al. (24) originally developed this questionnaire. Mei et al. (25) adapted it for China and confirmed the Chinese version’s reliability and validity among female breast cancer patients. This questionnaire primarily evaluated patients’ negative emotions toward disease, encompassing disease cognition, emotion, and disease understanding through 9 items spanning 3 dimensions. An 11-level scoring methodology (0–10) characterized this questionnaire, generating total scores ranging 0–80. Elevated scores reflected intensified negative emotional responses toward disease among patients. The present study yielded a Cronbach’s *α* coefficient of 0.87 for this questionnaire.

### 2.5 General self-efficacy scale (GSES)

Schwarzer et al. (26) originally developed this scale. Wang et al. (27) completed its Chinese translation. This single-dimension scale incorporated 10 items. Likert 4-level scoring methodology assigned scores spanning 1–4 points from completely incorrect to completely correct. Total scale scores ranged 10–40. Elevated scores indicated heightened individual self-efficacy. The present study obtained a Cronbach’s *α* coefficient of 0.87 for this scale.

### 2.6 Medical coping modes questionnaire (MCMQ)

Feifel et al. (28) originally developed this questionnaire. Shen and Jiang (29) completed its Chinese translation. This questionnaire incorporated 20 items across three dimensions: confronting, avoidance, and acceptance-resignation. Likert 4-level scoring methodology was employed. The confronting dimension generated total scores of 18–32, the avoidance dimension yielded total scores of 7–28, and the acceptance-resignation dimension produced scores of 5–20. The present study obtained Cronbach’s *α* coefficients of 0.94, 0.85, and 0.81 for confronting, avoidance, and acceptance-resignation, respectively.

### 2.7 Ethical considerations and data collection

The Institutional Review Board of XX University granted approval for this study (number: JZMULL2023047), ensuring adherence to ethical principles outlined in the Helsinki Declaration. Between November 2023 and March 2024, the study was performed at the First Affiliated Hospital of Jinzhou Medical University. Clinical data gathering commenced upon securing approval from the geriatric Department director, cardiology department director, and head nurse. Data acquisition occurred exclusively when patients had achieved clinical stability following treatment completion. Prior to formal data gathering, investigators clarified the investigation’s objectives and importance to participants, guaranteeing their comprehension of study protocols and their voluntary enrollment. Throughout questionnaire distribution, investigators employed one-to-one delivery methods. Immediate on-site collection of all questionnaires was performed, incomplete sections were addressed instantly, and questionnaires lacking validity underwent screening and removal.

### 2.8 Data analysis

Descriptive statistical analysis utilized SPSS25.0 software. Measurement data presentation employed mean and standard deviation, while counting data utilized frequency or component ratio. One-way ANOVA or *t*-test for single factors enabled comparison of demographic data’s impact on self-regulated fatigue. Pearson correlation analysis examined variable relationships. Multiple stepwise linear regression analysis identified factors influencing self-regulation fatigue. *p* < 0.05 is considered statistically significant. The variance inflation factor (VIF) was used to measure multicollinearity among independent variables in a multiple linear regression model. When VIF is less than 10, it is generally considered that there is no multicollinearity, indicating that the estimated regression coefficients of the model are relatively stable and the model is highly reliable. AMOS23.0 software facilitated structural equation model construction examining relationships among disease perception, self-efficacy, coping styles, and self-regulatory fatigue. Model fitting effectiveness and mediating effects underwent evaluation through Bootstrap methodology, with statistical significance established at *p* < 0.05. When the model fit indicators show that *χ*^2^/df is less than 3, RMSE is less than 0.05, and CFI, TLI, and GFI are greater than 0.90, it indicates that the model has a good fit.

## 3 Results

### 3.1 Demographic characteristics

The results of the descriptive statistical analysis indicated that the research data follow a normal distribution. This study enrolled 416 older adult patients with hypertension, averaging (70.97 ± 6.76) years of age, spanning 60–84 years, comprising 215 males (51.7%). Primary school education or lower characterized 32.5% of older adult hypertension patients, while 78.4% maintained married status. Findings indicated that advancing patient age correlated with increased self-regulatory fatigue severity, diminished educational attainment associated with heightened self-regulatory fatigue severity, extended hypertension duration corresponded to elevated self-regulatory fatigue severity, increased comorbidity numbers related to intensified self-regulatory fatigue, reduced per capita monthly household income linked to aggravated self-regulatory fatigue, and chronic pain presence in patients associated with amplified self-regulatory fatigue severity. The remaining data are shown in Table 1.

**Table 1:** Influence of different social demography factors on self-regulatory fatigue in older adult patients with hypertension.

Variables	*N* (%)	Score (*x̄* ± *s*)	Statistical value	*P*
Gender			*t* = −1.16	0.240
Male	215 (51.7%)	54.54 ± 14.91		
Female	201 (48.3%)	56.24 ± 14.88		
Age			*F* = 10.19	<0.001^*^
60 ~ 69	197 (47.4%)	51.97 ± 15.51		
70 ~ 79	177 (42.5%)	58.20 ± 13.84		
≥80	42 (10.1%)	59.31 ± 13.06		
Educational level			*F* = 40.98	<0.001^*^
Primary and below	135 (32.5%)	59.72 ± 11.67		
Junior high school	165 (39.6%)	58.15 ± 12.20		
High school or technical secondary school	90 (21.6%)	50.76 ± 16.75		
Bachelor degree or above	26 (6.3%)	31.00 ± 11.65		
Pre-retirement occupation			*F* = 2.15	0.093
Peasant	57 (13.7%)	58.14 ± 13.78		
Individual business	118 (28.4%)	54.18 ± 16.09		
Unit staff	229 (55.0%)	55.71 ± 14.52		
Other occupations	12 (2.9%)	47.25 ± 12.86		
Marital status			*F* = 0.65	0.580
Married	326 (78.4%)	55.71 ± 14.87		
Unmarried	2 (0.5%)	58.50 ± 13.44		
Divorced	25 (6.0%)	51.52 ± 15.17		
Widowed	63 (15.1%)	55.02 ± 15.15		
Per capita monthly household income (yuan)			*F* = 23.84	<0.001^*^
≤1,000	10 (2.4%)	66.30 ± 8.67		
1001–3000	180 (43.3%)	58.20 ± 12.63		
3001–5000	178 (42.8%)	55.95 ± 14.61		
>5,000	48 (11.5%)	40.27 ± 16.65		
Years of hypertension (years)			*F* = 23.86	<0.001*
<5	55 (13.2%)	42.33 ± 16.24		
5–10	128 (30.8%)	53.90 ± 15.58		
11–15	132 (31.7%)	58.68 ± 12.17		
≥16	101 (24.3%)	59.98 ± 12.04		
Number of complications (kinds of)			*F* = 35.99	<0.001*
None	130 (31.2%)	45.64 ± 16.26		
1	128 (30.8%)	57.46 ± 14.05		
2	136 (32.7%)	61.54 ± 9.44		
≥3	22 (5.3%)	62.41 ± 9.38		
Chronic pain			*F* = 8.07	<0.001*
Yes	157 (37.7%)	61.68 ± 9.34		
No	259 (62.3%)	51.53 ± 16.30		

*p < 0.005 indicates that the difference is statistically significant.

### 3.2 Descriptive statistical analysis of self-regulatory fatigue, disease perception, self-efficacy, and medical coping style

Self-regulatory fatigue scored 55.36 ± 14.91 among older adult hypertension patients in this study, indicating a medium-high level. Cognitive control, emotional control, and behavioral control registered scores of 20.59 ± 5.78, 17.72 ± 4.76, and 17.05 ± 4.89, respectively. Illness perception totaled 52.83 ± 13.63, representing a medium-high level. Self-efficacy achieved a total score of 25.46 ± 5.73, positioning at the middle level. Confrontation measured 13.02 ± 5.35, avoidance coping recorded 16.41 ± 4.57 and acceptance-resignation registered 12.05 ± 3.12. Detailed results are shown in Table 2.

**Table 2:** Scores of various scales in older adult patients with hypertension.

Variable	Number of items (n)	Range	Mean ± SD (*x̄* ± *s*)	Mean ± SD (*x̄* ± *s*) (each item)
Self-regulatory fatigue (total points)	16	16 ~ 80	55.36 ± 14.91	3.46 ± 0.93
Cognitive control	6	6 ~ 30	20.59 ± 5.78	3.43 ± 0.96
Emotional control	5	5 ~ 25	17.72 ± 4.76	3.54 ± 0.95
Behavior control	5	5 ~ 25	17.05 ± 4.89	3.41 ± 0.98
Disease perception	9	0 ~ 80	52.83 ± 13.63	6.60 ± 1.70
Disease cognition	5	0 ~ 50	33.30 ± 8.54	6.66 ± 1.71
Emotion	2	0 ~ 20	12.90 ± 4.17	6.45 ± 2.09
Disease understanding	1	0 ~ 10	6.63 ± 2.48	6.63 ± 2.48
Self-efficacy	10	10 ~ 40	25.46 ± 5.73	2.55 ± 0.57
confrontation	8	8 ~ 32	13.02 ± 5.35	1.63 ± 0.67
avoidance	7	7 ~ 28	16.41 ± 4.57	2.34 ± 0.65
Acceptance resignation	5	5 ~ 20	12.05 ± 3.12	2.41 ± 0.62

The values of all scales follow a normal distribution.

### 3.3 Correlations between variables

Pearson correlation analysis revealed positive associations between self-regulatory fatigue and illness perception (*r* = 0.68, *p* < 0.001), negative associations with self-efficacy (*r* = −0.63, *p* < 0.001) and confrontation (*r* = −0.49, *p* < 0.001), alongside positive associations with avoidance (*r* = 0.49, *p* < 0.001) and acceptance-resignation (*r* = 0.61, *p* < 0.001). Illness perception exhibited negative associations with self-efficacy (*r* = −0.40, *p* < 0.001) and confrontation (*r* = −0.32), while demonstrating positive associations with avoidance (*r* = 0.36, *p* < 0.001) and acceptance-resignation (*r* = 0.48, *p* < 0.001). Self-efficacy displayed positive associations with confronting coping (*r* = 0.34, *p* < 0.001), while showing negative associations with confrontation (*r* = −0.39, *p* < 0.001) and acceptance-resignation (*r* = −0.43, *p* < 0.001). The specific results are shown in Table 3.

**Table 3:** Correlation between self-regulatory fatigue and illness perception, self-efficacy, and coping style in older adult patients with hypertension.

	Self-regulatory fatigue	Disease perception	Self-efficacy	Confrontation	Avoidance	Acceptance-resignation
Self-regulatory fatigue	1.00					
Disease perception	0.68**	1.00				
Self-efficacy	−0.63**	−0.40**	1.00			
Confrontation	−0.49**	−0.32**	0.34**	1.00		
Avoidance	0.49**	0.36**	−0.39**	−0.16*	1.00	
Acceptance-resignation	0.61**	0.48**	−0.43**	−0.26**	0.41*	1.00

** means *P* < 0.001, * means *P* < 0.05.

### 3.4 Multiple linear regression analysis of influencing factors of self-regulated fatigue in older adult patients with hypertension

A multiple stepwise linear regression analysis identified self-regulatory fatigue among older adult patients with hypertension as the outcome variable (Y). Independent variables (X) comprised: age, educational attainment, monthly per capita household income, duration of hypertension, comorbidity count, chronic pain, disease perception, self-efficacy, confrontation, avoidance, and acceptance-resignation. The regression equation incorporated eight variables: illness perception, self-efficacy, acceptance-resignation, confrontation, educational attainment, comorbidity count, avoidance, and chronic pain (*F* = 148.39, *p* < 0.001). The *R*^2^ value attained 0.75, while adjusted *R*^2^ reached 0.74, explaining 74.0% of the total variation. A chain mediation pathway connected illness perception to self-regulatory fatigue through self-efficacy and coping style. The specific results are detailed in Table 4.

**Table 4:** Multiple linear regression analysis of self-regulated fatigue in older adult patients with hypertension.

Argument	*B*-value	Standard error	Beta	*t*-value	*P*-value	Tolerance	VIF
Constant	45.86	4.08	–	11.23	<0.001	–	–
Disease perception	0.33	0.03	0.30	9.77	<0.001	0.64	1.56
Self-efficacy	−0.63	0.08	−0.24	−7.85	<0.001	0.65	1.52
Acceptance-resignation	0.94	0.14	0.19	6.35	<0.001	0.65	1.53
Confrontation	−0.50	0.07	−0.18	−6.54	<0.001	0.81	1.22
Avoidance	0.39	0.09	0.12	4.19	<0.001	0.75	1.32
Education level	−1.87	0.45	−0.11	−4.14	<0.001	0.85	1.16
Number of complications	1.63	0.44	0.10	3.64	<0.001	0.82	1.21
Chronic pain	−1.88	0.82	−0.06	2.28	<0.001	0.87	1.14

*R*^2^ = 0.75, adjusted *R*^2^ = 0.74, *F* = 148.39, *P* < 0.001.

**Figure 1:**
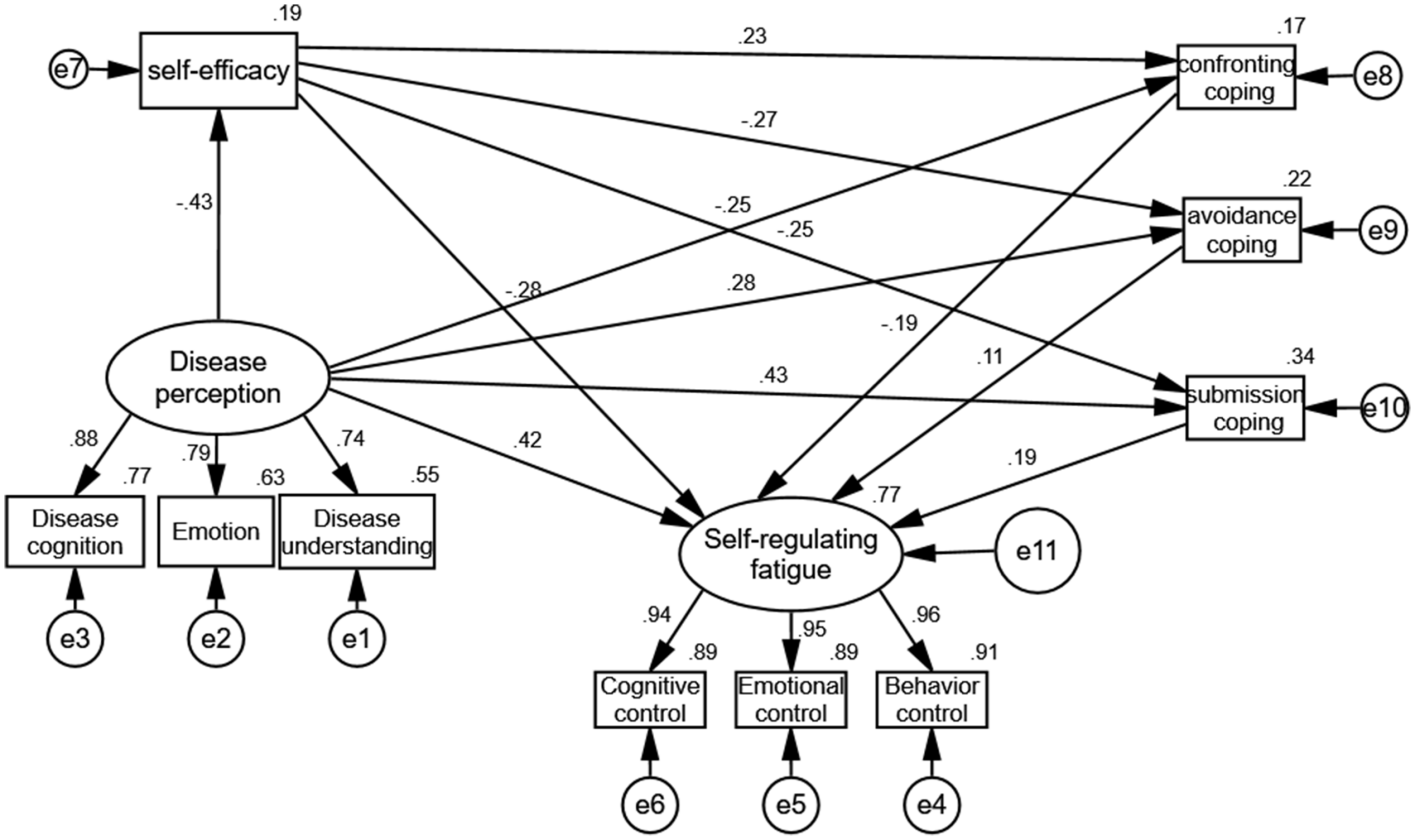
Final model path and standardized regression coefficients.

Within this study, illness perception operated as an independent variable, featuring three dimensions (illness cognition, emotion, and illness understanding) that served as latent variables. The mediating variables comprised confrontation, avoidance, and acceptance-resignation. Self-regulatory fatigue constituted the dependent variable, with its three dimensions (cognitive control, emotional control, and behavioral control) functioning as latent variables. Construction of the structural equation model utilized Amos23.0 software (Figure 1).

The maximum likelihood method facilitated model fitting, yielding statistical significance across all path differences (*p* < 0.001). Model fitting indexes remained within acceptable parameters, warranting model acceptance (Table 5). Illness perception exerted direct positive predictive effects on self-regulatory fatigue (*β* = 0.42, *p* < 0.001). Self-efficacy displayed direct negative predictive effects on self-regulatory fatigue (*β* = −0.27, *p* < 0.001). Confrontation showed direct negative predictive effects on self-regulatory fatigue (*β* = −0.19, *p* < 0.001). Avoidance exhibited direct negative predictive effects on self-regulatory fatigue (*β* = 0.11, *p* < 0.001). Acceptance-resignation manifested direct negative predictive effects on self-regulatory fatigue (*β* = 0.18, *p* < 0.001). Self-efficacy operated as a partial mediator between illness perception and self-regulatory fatigue. Confrontation functioned as a partial mediator connecting illness perception to self-regulatory fatigue; confrontation acted as a partial mediator linking illness perception to self-regulatory fatigue. Acceptance-resignation served as a partial mediator between illness perception and self-regulatory fatigue. Disease perception, confrontation, avoidance, and acceptance-resignation constituted chain mediators connecting illness perception to self-regulatory fatigue. The specific results are shown in Table 6.

**Table 5:** Model fitting index of self-regulatory fatigue in older adult patients with hypertension.

	*x* ^2^	df	*X*^2^/df	RMSEA	GFI	AGFI	CFI	NFI
Model index	63.31	27	2.34	0.05	0.97	0.94	0.98	0.97
References index			<3	<0.08	>0.90	>0.90	>0.90	>0.90

All fitting indicators are within the reference range.

**Table 6:** Direct effect, indirect and total effect of variables (standardized).

Effect	Path	*β*	Effect size (*n* %)	95%CI
Direct effect	Disease perception → Self-regulatory fatigue	0.42	55.82	0.35 ~ 0.49
Indirect effect	Disease perception → self-efficacy → Self-regulatory fatigue	0.30	39.95	0.22 ~ 0.40
	Disease perception → confrontation → Self-regulatory fatigue	0.12	16.40	0.06 ~ 0.20
	Disease perception → avoidance → Self-regulatory fatigue	0.08	10.85	0.03 ~ 0.14
	Disease perception → acceptance-resignation→ Self-regulatory fatigue	0.20	26.85	0.13 ~ 0.29
	Disease perception → self-efficacy → confrontation → Self-regulatory fatigue	0.05	6.61	0.02 ~ 0.08
	Disease perception → self-efficacy → avoidance → Self-regulatory fatigue	0.03	4.50	0.01 ~ 0.06
	Disease perception → self-efficacy → acceptance-resignation → Self-regulatory fatigue	0.05	6.88	0.02 ~ 0.08
Total effect	–	0.75	–	0.70 ~ 0.80

The total effect illustrates the overall impact of each independent variable on the dependent variable.

## 4 Discussion

Within older adult patients with hypertension, the present study examined existing associations among disease perception, self-efficacy, coping style, and self-regulatory fatigue. Furthermore, self-efficacy and coping style were demonstrated to exert a chain mediating effect in the association from illness perception to self-regulatory fatigue.

### 4.1 Effect of demographic characteristics on self-regulation fatigue in older adult patients with hypertension

This study identified educational attainment, comorbidity count, and chronic pain as determinants of self-regulatory fatigue among older adult patients with hypertension. Higher levels of education were associated with lower levels of self-regulatory fatigue, which is consistent with what Gao et al. (30) found. Patients with higher levels of education exhibit greater empowerment and self-control, and as a result, self-regulatory fatigue is reduced (31). Patients with higher comorbidity rates are under more psychological stress due to increased clinical symptoms and more physical challenges that need to be overcome, which leads to increased resource use and exhaustion during self-management (32). Patients with chronic pain in this study had comparatively high levels of self-regulatory fatigue. This is in line with what Haramaki et al. (33) found, which may be explained by the unpredictable and uncontrollable nature of pain, since patients are required to continuously develop negative pain expectations during this process, which results in significant psychological strain (34). To mitigate anxiety and depression resulting from chronic pain, patients devote greater energy to self-regulatory, leading to elevated self-regulatory fatigue probability. Consequently, nursing staff in future practice ought to emphasize how educational attainment, comorbidity count, and chronic pain affect self-regulatory fatigue among hypertensive older adults, and develop individualized intervention strategies based on patients’ general condition and disease characteristics. For patients with low literacy, it should gradually popularize disease-related knowledge to patients, supplemented by psychological counseling, so that patients can understand the important role of mental health in disease management and reduce their psychological fatigue. For patients with multiple comorbidities, it is necessary to start from the actual condition to alleviate the patient’s pain, and encourage patients with certain physical activity ability to exercise more to improve their physical fitness, communicate more with patients, reduce their fear of having multiple comorbidities, and enhance their confidence. Patients with chronic pain should be treated symptomatically to reduce their pain. In addition, the caregiver can also play some relaxing music for the patient to distract them and give them enough companionship and comfort.

### 4.2 Analysis of self-regulatory fatigue, disease perception, self-efficacy and coping style in older adult patients with hypertension

The present study’s outcomes revealed that self-regulatory fatigue levels among older adult patients with hypertension exceeded domestic normative values, positioning them at an elevated level (35). This signified that older patients with hypertension exhibit pronounced self-regulatory fatigue, potentially attributable to diminished self-management capacity stemming from prolonged hypertension duration, suboptimal blood pressure regulation outcomes, and extended medication periods, thereby generating psychological burden among patients and associating with self-regulatory fatigue (36). As a chronic illness, hypertension has a poor prognosis, low control rates, and many complications for older patients. In addition to physical pain, medical costs, the risk of complications, and inadequate family support have additional effects on the mental health of older adults with hypertension during the prolonged course of treatment (37, 38). Long-term deterioration of the patient’s capacity for self-control and self-regulatory of the negative effects of the disease results in self-regulatory fatigue. This highlighted the need for nursing staff to continuously monitor patients’ psychological exhaustion during clinical practice, promptly inform patients about the disease, show successful hypertension control cases, stress the importance of self-management, and help patients reduce their psychological burden.

Within the present study, illness perception scores among older adults with hypertension reached higher levels, surpassing those from Alfian et al.’s (39) survey examining illness perception among older adults with hypertension. This implied that older adults with hypertension possess elevated negative disease emotions. This may correlate with the fact that the present study examined hospitalized older hypertensive patients, the majority experiencing multiple diseases and lacking capability to adequately judge and evaluate their disease status (40). Additionally, older adults undergo aging effects, memory deterioration, and progressive degradation across various bodily functions, resulting in their incapacity to comprehensively grasp disease mechanisms, absence of pertinent disease knowledge experience, and tendency toward elevated negative emotions concerning current disease situations (41, 42). Consequently, nursing staff should intensify psychological counseling for older hypertension patients throughout clinical work, and transform their fixed mindsets and erroneous disease cognition.

Within the present study, self-efficacy among middle-aged and older adults with hypertension remained at moderate levels, aligning with Lu et al.’s (43) outcomes. A negative correlation existed between self-efficacy and self-regulatory fatigue among older adults with hypertension (*p* < 0.001), demonstrating that elevated patient self-efficacy corresponds with reduced self-regulatory fatigue degrees, concordant with prior study results (44). This comprehensively reflected individual belief’s vital function throughout disease management processes, which effectively diminishes patients’ psychological resource consumption and reduces patients’ self-regulatory fatigue. Accordingly, nursing staff should strengthen hypertensive patients’ belief against disease, validate their personal capabilities, and facilitate patients in establishing confidence for overcoming disease.

The present study identified confrontation as a protective element of self-regulatory fatigue, while avoidance and acceptance-resignation represented risk elements of self-regulatory fatigue. Within the present study, middle-aged and older adults with hypertension demonstrated greater inclination toward avoidant coping styles. This suggested that medical staff should promote patients adopting constructive approaches for addressing disease, and fulfill constructive functions in self-regulatory fatigue protection.

### 4.3 Self-regulatory of the relationship between fatigue, disease perception, self-efficacy, and coping style

In contrast to self-efficacy and confrontation, which showed inverse relationships with self-regulatory fatigue, this study found positive associations between self-regulatory fatigue and disease perception, avoidance, and acceptance-resignation. This showed that patients’ increased negative emotional reactions to their perception of the disease are correlated with elevated levels of self-regulatory fatigue. Therefore, scientific disease knowledge should be applied throughout nursing practice to help patients reduce negative emotional states and negative disease perceptions. Concurrently, it is critical to acknowledge the critical role that self-belief plays in managing illness, boost patient confidence in the course of treatment, and support the development of patients’ self-regulatory skills in order to reduce self-regulatory fatigue. While avoidance and acceptance-resignation styles weaken patients’ ability to exercise self-control, resulting in self-regulatory fatigue when managing the negative effects of various diseases, constructive coping strategies help patients feel less depressed (45). Nursing staff should therefore create focused intervention plans based on the ways that self-efficacy, coping style, and illness emotion affect self-regulatory fatigue.

### 4.4 The role of self-efficacy and coping style in the chain mediation between illness perception and self-regulatory fatigue

Mediating effect outcomes revealed that illness perception exerted not only direct positive influence upon self-regulatory fatigue, but also impacted self-regulatory fatigue via chain mediating pathways involving self-efficacy coupled with confrontation, self-efficacy paired with avoidance, and self-efficacy combined with acceptance-resignation. This potentially stems from disease perception’s adverse impact on self-efficacy. Elevated negative emotional responses toward disease among patients diminish their treatment belief, prompting them to select avoidance or submission toward disease, consequently elevating self-regulatory fatigue (16). This is consistent with the concept proposed in the self-regulatory fatigue model: when individuals perceive a threat to their health or illness, they first conduct self-assessment and then adopt different coping strategies, ultimately leading to fatigue in an endless cycle. Furthermore, illness perception demonstrated adverse effects on confrontation alongside positive effects on avoidance and acceptance-resignation. This potentially originates from patients’ reluctance to address disease-related challenges when perceiving negative emotions generated by disease, leading them to opt for avoidance or surrender. Under these circumstances, patient self-regulatory capacity declines, triggering fatigue. The present study’s outcomes additionally validate the self-regulatory model’s conceptual framework. Thus, throughout clinical work, while monitoring disease perception’s direct influence on self-regulatory fatigue, clarifying self-efficacy and coping style impacts on patients’ self-regulatory conditions becomes equally necessary.

## 5 Conclusion

Self-regulation fatigue receives direct positive influence from illness perception. Self-regulatory fatigue is additionally affected by illness perception through chain mediating pathways involving self-efficacy combined with confrontation, self-efficacy combined with avoidance, and self-efficacy combined with acceptance-resignation. Self-regulatory fatigue concepts were first introduced to older adult patients with hypertension in the present study, providing theoretical foundations for nursing staff in developing intervention strategies. In future nursing practice, nursing staff ought to fully utilize self-efficacy’s protective influence on self-regulatory fatigue, encourage patients to actively confront their disease, strengthen psychological, emotional, and cognitive interventions for patients, timely modify self-regulatory fatigue status in hypertension patients, consequently improve patients’ disease self-management capability, and more effectively promote disease treatment progression for hypertension patients.

### 5.1 Limitations

As this study was performed in merely one hospital ranked among the top three facilities within a single Chinese city, broader applicability of findings necessitates additional verification. Subsequent studies should expand the sample size and scope of study populations, incorporating older adults individuals with hypertension from community settings to perform multi-center cross-sectional investigations. Additionally, employing a longitudinal study will address the inherent limitations of the cross-sectional study approach. The scales used in this study were self-assessment tools, which may lead to certain biases in the research results. Based on the existing study findings, there may be unmeasured influencing factors in this study. In future research, more literature findings should be considered when selecting the influencing factors to be included in the study.

## Data Availability

The raw data supporting the conclusions of this article will be made available by the authors, without undue reservation.

## References

[1] Yin R Yin L Li L Silva-Nash J Tan J Pan Z . Hypertension in China: burdens, guidelines and policy responses: a state-of-the-art review. J Hum Hypertens. (2022) 36:126–34. doi: 10.1038/s41371-021-00570-z, 34215840 PMC8252986

[2] Guasti L Ambrosetti M Ferrari M Marino F Ferrini M Sudano I . Management of Hypertension in the elderly and frail patient. Drugs Aging. (2022) 39:763–72. doi: 10.1007/s40266-022-00966-7, 35904720 PMC9553775

[3] Pont L Alhawassi T. Challenges in the management of hypertension in older populations. Adv Exp Med Biol. (2017) 956:167–80. doi: 10.1007/5584_2016_149, 27815929

[4] Zhang XN Qiu C Zheng YZ Zang XY Zhao Y. Self-management among elderly patients with hypertension and its association with individual and social environmental factors in China. J Cardiovasc Nurs. (2020) 35:45–53. doi: 10.1097/JCN.0000000000000608, 31373957

[5] Baumeister RF Bratslavsky E Muraven M Tice DM. Ego depletion: is the active self a limited resource? J Pers Soc Psychol. (1998) 74:1252–65. doi: 10.1037//0022-3514.74.5.1252, 9599441

[6] Nes LS Ehlers SL Whipple MO Vincent A. Self-regulatory fatigue in chronic multisymptom illnesses: scale development, fatigue, and self-control. J Pain Res. (2013) 6:181–8. doi: 10.2147/JPR.S40014, 23526193 PMC3596127

[7] Li X Gao Q Sun L Gao W. Effect of self-control on health promotion behavior in patients with coronary heart disease: mediating effect of ego-depletion. Psychol Health Med. (2022) 27:1268–76. doi: 10.1080/13548506.2020.1867316, 33813978

[8] Hansen MH Primdahl J Riber L Ekholm O Kristensen KL Thrysoee L . Illness perception after heart valve surgery: differences among men and women. J Cardiovasc Nurs. (2021) 36:329–39. doi: 10.1097/JCN.0000000000000676, 32379164

[9] Maninet S Desaravinid C. Relationships between illness perception, functional status, social support, and self-care behavior among thai people at high risk of stroke: a cross-sectional study. Belitung Nurs J. (2023) 9:62–8. doi: 10.33546/bnj.2434, 37469636 PMC10353622

[10] Xiong C Jiang C Zhang H Chen J Zhao M Xiong C . Self-management and illness perception among cervical cancer patients: a cross-sectional study. Int J Nurs Pract. (2023) 29:e13134. doi: 10.1111/ijn.13134, 36708017

[11] Bandura A. Social cognitive theory: an agentic perspective. Annu Rev Psychol. (1999) 52:1–26. doi: 10.1111/1467-839X.00024 11148297

[12] Yu H Yang L Tian J Austin L Tao Y. The mediation role of self-control in the Association of Self-Efficacy and Physical Activity in college students. Int J Environ Res Public Health. (2022) 19:12152. doi: 10.3390/ijerph191912152, 36231454 PMC9564918

[13] Kurt S Altan SN. Correlation of self-efficacy and symptom control in cancer patients. Support Care Cancer. (2022) 30:5849–57. doi: 10.1007/s00520-022-06972-0, 35364732

[14] Li H Chang H Tao Z Zhang D Shi Y Li X. Mediating effect of coping style on the relationship between clinical leadership and quality of work life among nurses in tertiary-level hospitals in China: a cross-sectional study. BMJ Open. (2021) 11:e041862. doi: 10.1136/bmjopen-2020-041862, 33597134 PMC7893656

[15] Wang X Sheng Y. Readiness for advance care planning and its relationship to coping style in patients with chronic diseases in communities: a cross-sectional study. Nurs Open. (2022) 9:1332–42. doi: 10.1002/nop2.1178, 35092182 PMC8859049

[16] Liu Q Mo L Huang X Yu L Liu Y. Path analysis of the effects of social support, self-efficacy, and coping style on psychological stress in children with malignant tumor during treatment. Medicine (Baltimore). (2020) 99:e22888. doi: 10.1097/MD.0000000000022888, 33120834 PMC7581179

[17] Lucke HR Carey CN Griffith EL Mathes EW Lane DJ Boals A. Self-control, coping styles, and alcohol outcomes in college students. J Am Coll Heal. (2024) 72:3376–83. doi: 10.1080/07448481.2022.2160260, 36701421

[18] Leventhal H Phillips LA Burns E. The common-sense model of self-regulation (CSM): a dynamic framework for understanding illness self-management. J Behav Med. (2016) 39:935–46. doi: 10.1007/s10865-016-9782-2, 27515801

[19] Inzlicht M Werner KM Briskin JL Roberts BW. Integrating models of self-regulation. Annu Rev Psychol. (2021) 72:319–45. doi: 10.1146/annurev-psych-061020-105721, 33017559

[20] Writing Group of 2018 Chinese Guidelines for the Management of Hypertension, Chinese Hypertension League, Hypertension Branch of China International Exchange and Promotive Association for Medical and Health Care . 2024 Chinese guidelines for the management of hypertension. Chin J Hypertens. (2024) 32:606–7. doi: 10.16439/j.issn.1673-7245.2024.07.002

[21] Kendall MG. Multivariate analysis. London: Griffin, Duxbury (1975). 210 p.

[22] Jak S Cheung MW. Meta-analytic structural equation modeling with moderating effects on SEM parameters. Psychol Methods. (2020) 25:430–55. doi: 10.1037/met0000245, 31670537

[23] Wang L Zhang JY Wang J Tao T Fan CL Gao WB. The Chinese version of self-regulated fatigue scale was used to evaluate the validity and reliability of young people. Chin Ment Health J. (2015) 4:290–4. doi: 10.3969/j.issn.1000-6729.2015.04.010, (Chinese)

[24] Broadbent E Petrie KJ Main J Weinman J. The brief illness perception questionnaire. J Psychosom Res. (2006) 60:631–7. doi: 10.1016/j.jpsychores.2005.10.020, 16731240

[25] Mei YQ Li HP Yang YJ Su D Ma L Zhang T . Reliability and validity of simplified version of Chinese version of disease perception questionnaire in female breast cancer patients. J Nurs. (2015) 22:11–4. doi: 10.16460/j.issn1008-9969.2015.24.011, (Chinese)

[26] Schwarzer R Born A. Optimistic self-beliefs: assessment of general perceived self-efficacy in thirteen cultures. World Psychol. (1997) 3:177–90.

[27] Wang CK Hu ZF Liu Y. Reliability and validity of general self-efficacy scale. J Appl Psychol. (2001) 7:37–40. doi: 10.3969/j.issn.1006-6020.2001.01.007

[28] Feifel H Strack S Nagy VT. Coping strategies and associated features of medically ill patients. Psychosom Med. (1987) 49:616–25. doi: 10.1097/00006842-198711000-00007, 3423168

[29] Shen XH Jiang QJ. Chinese version of the medical coping style questionnaire: a report of 701 cases. J Behav Med Brain Sci. (2000) 1:18–20.

[30] Gao Y Shan Y Jiang T Cai L Zhang F Jiang X . Dietary adherence, self-regulatory fatigue and trait self-control among Chinese patients with peritoneal dialysis: a cross-sectional study. Patient Prefer Adherence. (2021) 15:443–51. doi: 10.2147/PPA.S298231, 33658768 PMC7920602

[31] Zhang Y He H Yang C Wang X Luo J Xiao J . Chain mediations of perceived social support and emotional regulation efficacy between role stress and compassion fatigue: insights from the COVID-19 pandemic. Front Public Health. (2023) 11:1269594. doi: 10.3389/fpubh.2023.1269594, 38026273 PMC10680973

[32] Li SH Lloyd AR Graham BM. Physical and mental fatigue across the menstrual cycle in women with and without generalised anxiety disorder. Horm Behav. (2020) 118:104667. doi: 10.1016/j.yhbeh.2019.104667, 31899259

[33] Haramaki Y Kabir RS Abe K Yoshitake T. Promoting self-regulatory Management of Chronic Pain through Dohsa-hou: single-case series of low-functioning Hemodialysis patients. Front Psychol. (2019) 10:1394. doi: 10.3389/fpsyg.2019.01394, 31281283 PMC6596355

[34] Barakou I Hackett KL Finch T Hettinga FJ. Self-regulation of effort for a better health-related quality of life: a multidimensional activity pacing model for chronic pain and fatigue management. Ann Med. (2023) 55:2270688. doi: 10.1080/07853890.2023.2270688, 37871249 PMC10595396

[35] Cui Y Li R Yang T Wang H Jin S Liu N . Influence of positive and negative affect on self-management among patients with early chronic kidney disease during the COVID-19 pandemic: the mediating and suppressing effect of ego depletion. Front Psych. (2022) 13:992404. doi: 10.3389/fpsyt.2022.992404, 36245863 PMC9556950

[36] Bosworth HB Powers BJ Oddone EZ. Patient self-management support: novel strategies in hypertension and heart disease. Cardiol Clin. (2010) 28:655–63. doi: 10.1016/j.ccl.2010.07.003, 20937448 PMC3763915

[37] Rogers EA Abi H Linzer M Eton DT. Treatment burden in people with hypertension is correlated with patient experience with self-management. J Am Board Fam Med. (2021) 34:1243–5. doi: 10.3122/jabfm.2021.06.210191, 34772780 PMC9110114

[38] Turana Y Tengkawan J Chia YC Shin J Chen CH Park S . Mental health problems and hypertension in the elderly: review from the HOPE Asia network. J Clin Hypertens. (2021) 23:504–12. doi: 10.1111/jch.14121, 33283971 PMC8029564

[39] Alfian SD Annisa N Perwitasari DA Coelho A Abdulah R. The role of illness perceptions on medication non-adherence among patients with hypertension: a multicenter study in Indonesia. Front Pharmacol. (2022) 13:985293. doi: 10.3389/fphar.2022.985293, 36225558 PMC9549155

[40] Shi W Cheng L Li Y. Influence of "hospital-community-family" integrated management on blood pressure, quality of life, anxiety and depression in hypertensive patients. Comput Math Methods Med. (2022) 2022:1962475. doi: 10.1155/2022/1962475, 36238498 PMC9553346

[41] Khan ZU Martín-Montañez E Navarro-Lobato I Muly EC. Memory deficits in aging and neurological diseases. Prog Mol Biol Transl Sci. (2014) 122:1–29. doi: 10.1016/B978-0-12-420170-5.00001-5, 24484696

[42] Juan SMA Adlard PA. Ageing and cognition. Subcell Biochem. (2019) 91:107–22. doi: 10.1007/978-981-13-3681-2_5, 30888651

[43] Lu T Yang Z Chen P Li J Zheng C Kong L . Influencing factors of medication literacy among community-dwelling older adult patients with hypertension: a study based on social learning theory. Front Pharmacol. (2023) 14:1184701. doi: 10.3389/fphar.2023.1184701, 37332350 PMC10272614

[44] Li L Liu H Wang G Chen Y Huang L. The relationship between ego depletion and prosocial behavior of college students during the COVID-19 pandemic: the role of social self-efficacy and personal belief in a just world. Front Psychol. (2022) 13:801006. doi: 10.3389/fpsyg.2022.801006, 35548506 PMC9083063

[45] Boals A Vandellen MR Banks JB. The relationship between self-control and health: the mediating effect of avoidant coping. Psychol Health. (2011) 26:1049–62. doi: 10.1080/08870446.2010.529139, 21598180

